# Editorial: Carbon monoxide poisoning: updates on prevention, diagnosis, and treatment

**DOI:** 10.3389/fmed.2024.1411547

**Published:** 2024-04-16

**Authors:** Matteo Paganini, Stephen R. Thom

**Affiliations:** ^1^Department of Biomedical Sciences, University of Padova, Padova, Italy; ^2^Department of Emergency Medicine, School of Medicine, University of Maryland, Baltimore, MD, United States

**Keywords:** carbon monoxide, toxicology, emergency medicine, intensive care, hyperbaric medicine, oxygen

Carbon Monoxide (CO). This name will ring a bell in the reader's mind as a gas that is poisonous, colorless, and odorless. Also, one will recall that CO intoxication is challenging to diagnose due to these physical properties and to the unspecific symptoms and clinical signs manifested, often overlapping with several potential other diseases. With this Research Topic titled “*Carbon monoxide poisoning: updates on prevention, diagnosis, and treatment*,” we gathered updates and improvements in the abovementioned fields. Ten manuscripts were submitted, and five were accepted: two discussing the “diagnosis” field, two with a prognostic perspective, and one discussing CO intoxication treatment. Overall, it is unfortunate that public health aspects dealing with “prevention” have not attracted a submission, which we hope for the future.

The diagnosis of CO intoxication is an interplay among clinical presentation, biomarkers, and a suspect CO source found in the location where the intoxication occurred. Unfortunately, each element has significant limitations. First, there is low awareness among clinicians, which critically impairs accurate and timely diagnosis (1). Moreover, a wide range of symptoms have been described in CO poisoning, from a mild headache (the most prevalent) to coma and death. The suspicion of intoxication may arise if the situation is suggestive of CO generation, e.g., from fires or heat generators in poorly ventilated spaces. However, rescue teams may not identify such intoxication sources or inconsistently report them to clinicians. Patients may also present to health care workers autonomously with symptoms but without reporting a suspected source or etiology, making the diagnosis more difficult.

Oliverio has summarized the state of the art regarding diagnostic biomarkers from an “analytical perspective.” The limitations of current CO measurement techniques have been described, particularly carboxyhemoglobin (CO-Hb), which is the most used marker but is influenced by smoking habits and, on occasion, environmental pollution. One must also remember that CO-Hb values diminish when the patient is removed from the CO source. Therefore, a normal CO-Hb does not exclude CO poisoning. Moreover, CO-Hb does not have the potential to inform regarding levels of CO intoxication at a cellular level. The proposed new biomarker is the total blood carbon monoxide (TBCO), apparently offering a more reliable indicator whose application in emergency medicine should be further studied (primarily limited by the current analytical technique requiring gas chromatography–mass spectrometry, which is not readily available in all laboratories).

The systematic review and meta-analysis published by Ramponi et al. enriched the diagnostic side of this topic, aiming to shed light on the accuracy of a commercially available CO pulse oximetry device. After including six studies, the authors concluded that despite its high specificity, CO pulse oximetry does not have sufficient sensitivity to be systematically used in clinical practice as a screening tool. Of note, the authors acknowledged the variability of the CO-Hb cutoff to define a patient as intoxicated and set the threshold to the upper limit of 10%. The authors suggested studying this device in the prehospital arena, especially when rescuing multiple casualties after fires or mass intoxications, or for identifying subtle intoxications to directly access a hospital with hyperbaric oxygen (HBO_2_) treatment capability (2).

On the other hand, the interest of the other three papers focused on Delayed Neurologic Sequelae (DNS), a poorly understood condition that may occur as late as 2–6 weeks after CO intoxication. DNS manifestations include neuropsychiatric symptoms such as cognitive deterioration, sphincters incontinence, a parkinsonism-like condition, akinetic mutism, dystonia, neurological focal symptoms, and depression/anxiety (3–5). The exact mechanism of DNS development and its incidence are still to be defined (6). A significant problem is represented by a lucid interval between the clinical encounter and DNS development, frequently misleading the recognition and hampering correct treatment. Cha et al. analyzed and confirmed the superiority of Quantitative Pupillary Reflex (qPLR) and neurological pupil index (NPi) measured during the first 2 days of admission against standard Pupillary Light Reflex (nPR) in predicting poorer neurocognitive outcomes 1 month after CO poisoning on a sample of 104 adults. Gao et al. presented a single-center retrospective analysis of adult patients diagnosed with CO poisoning, comparing the characteristics of 25 adult patients who developed DNS vs. 48 who did not manifest neuropsychiatric symptoms in the follow-up period (6 weeks from discharge). The findings identified a longer duration of CO exposure (especially if more than 5.5 h) and the presence of acute brain lesions on diffusion-weighted imaging within 24 h as independent risk factors for DNS. Overall, these 2 papers provide some indicators potentially helpful for identify patients at risk of more serious DNS. Further studies are needed to determine whether these patients may benefit from a prolonged observation in the emergency department, admission to the ward, and more timely or intense rehabilitative therapy.

Also, the best treatment for DNS is still debated, especially regarding the number of HBO_2_ treatments, the exact pressures and duration to be used, and whether HBO_2_ should be administered alone or with adjuvants such as antioxidant scavengers or corticosteroids (6). Lee et al. prospectively analyzed data collected from a registry established in 2006, including 537 patients, and found no differences in the reduction of poor neurocognitive outcomes among patients who received one, two, or three HBO_2_ sessions within 24 h after CO poisoning. The authors suggested that one HBO_2_ treatment within 24 h may be reasonable, but more trials should be performed before issuing high-level recommendations.

Overall, the submitted studies provided interesting insights and advancements in the niche of CO intoxication. We hope that such new information can promote discussion among the involved healthcare providers to improve the quality of care, starting from prevention through diagnosis and considerations of the best treatment(s) to preserve the quality of life of CO-intoxicated patients.

## Author contributions

MP: Conceptualization, Visualization, Writing – original draft, Writing – review & editing. ST: Conceptualization, Supervision, Visualization, Writing – original draft, Writing – review & editing.

## References

[1] Malik FS Asswad RG Clarke S. Carbon monoxide: raising awareness of the silent killer in the emergency department. BMJ Open Qual. (2022) 11:e001777. 10.1136/bmjoq-2021-001777 35675945 PMC9185569

[2] Martani L Cantadori L Paganini M Camporesi EM Bosco G. Carbon monoxide intoxication: prehospital diagnosis and direct transfer to the hyperbaric chamber. Minerva Anestesiol. (2019) 85:6. 10.23736/S0375-9393.19.13648-6 30945521

[3] Hampson NB Hauff NM. Risk factors for short-term mortality from carbon monoxide poisoning treated with hyperbaric oxygen.^*^ *Critic Care Med*. (2008) 36:2523–7. 10.1097/CCM.0b013e31818419d8 18679118

[4] Hampson NB Piantadosi CA Thom SR Weaver LK. Practice recommendations in the diagnosis, management, and prevention of carbon monoxide poisoning. Am J Respir Crit Care Med. (2012) 186:1095–101. 10.1164/rccm.201207-1284CI 23087025

[5] Thom SR Taber RL Mendiguren II Clark JM Hardy KR Fisher AB. Delayed neuropsychologic sequelae after carbon monoxide poisoning: prevention by treatment with hyperbaric oxygen. Ann Emerg Med. (1995) 25:474–80. 10.1016/S0196-0644(95)70261-X 7710151

[6] Martani L Giovanniello A Bosco G Cantadori L Calissi F Furfaro D. Delayed neurological sequelae successfully treated with adjuvant, prolonged hyperbaric oxygen therapy: review and case report. IJERPH. (2022) 19:5300. 10.3390/ijerph19095300 35564694 PMC9104642

